# Analytical methods for determination of bisphenol A, 4-*tert*-octylphenol and 4-nonylphenol in herrings and physiological fluids of the grey seal

**DOI:** 10.1016/j.mex.2018.09.007

**Published:** 2018-09-21

**Authors:** Marta Staniszewska, Iga Nehring, Lucyna Falkowska, Karina Bodziach

**Affiliations:** Institute of Oceanography, University of Gdansk, Al. Marszałka Piłsudskiego 46, 81-378 Gdynia, Poland

**Keywords:** Determination of bisphenol A, 4-*tert*-octylphenol and 4-nonylphenol in whole fish, blood and milk samples by HPLC-FL, Phenol derivatives, Biological samples, Liquid chromatography, Fluorescence detector

## Abstract

• The aim of this work was to develop the methods of determination for phenol derivatives: bisphenol A (BPA), 4-*tert*-octylphenol (OP) and 4-nonylphenol (NP), in the whole body of herring *Clupea harengus* and in physiological fluids of the Baltic grey seal *Halichoerus grypus grypus* (blood and milk).
• Methods were based on liquid chromatography coupled with a fluorescence detector (HPLC-FL).
• These methods were satisfactorily validated, each showing good recovery (>80%) and precision (RSD < 15%). Regarding the limit of quantification (LOQ), this was established at <2 ng g^−1^ for herring, <0.07 ng cm^-3^ for blood and <0.1 ng cm^−3^ for milk.

## Highlights

• These methodologies have three key benefits.
• Simultaneous determination of three endocrine disrupting phenol derivatives.
• High sensitivity - detection by fluorescence detector.
• Simply, short and not costly determination in blood and milk.

## Graphical abstract

**Figure:**
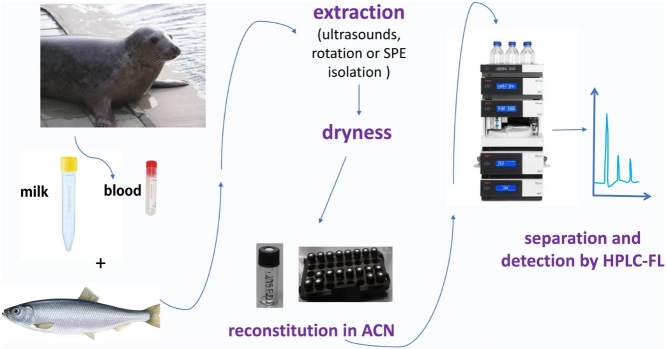

Specifications Table

**Table:** 

Subject area	• *Chemistry* • *Environmental Science* • *Immunology and Microbiology*
More specific subject area	biological samples: whole body of herring, physiological fluids of grey seal (blood and milk)
Method name	Determination of bisphenol A, 4-*tert-*octylphenol and 4-nonylphenol in whole fish, blood and milk samples by HPLC-FL
Name and reference of original method	Yi B, Kim C, Yang M, 2010. Biological monitoring of bisphenol A with HLPC/FLD and LC/MS/MS assays. Journal of Chromatography, (27):2606-10 Xiao Q, Li Y, Ouyang H, Xu P, Wu D (2006) High-performance liquid chromatographic analysis of bisphenol A and 4-nonylphenol in serum, liver and testis tissues after oral administration to rats and its application to toxicokinetic study. J. Chromatogr. B. Analyt. Technol. Biomed. Life Sci. 830:322-9
Resource availability	–

The aim of the conducted research was to develop a method allowing to determine at one analysis the concentrations of bisphenol A, 4-tert-octylphenol and 4-nonylphenol in biological tissues (samples of fish and physiological fluids of seals). All steps to carry out the determinations are described in detail in the Methods section.

## Method details

The methods developed in this work were applied to investigate the presence of selected contaminants: bisphenol A (BPA), 4*-tert*-octylphenol (OP) and 4-nonylphenol (NP) in the whole body of herring and in physiological fluids of the Baltic grey seal *Halichoerus grypus* (blood and milk). For this purpose, we used the available methods described earlier by Xiao et al [1] for rat tissues and blood, and by Yi et al. [2] for human milk. The novelty of the present paper is the extension of the above methods in order to assay a wider range of phenol derivatives. In the cases of tissues and blood, analytical methods were developed by 4-*tert*-octylphenol, and in the case of milk by 4-tert-octylphenol and 4-nonylphenol. The methods were also adopted for samples of different quality, i.e. ones originating from different organisms. The most significant differences can be visible between human milk and seal milk. The latter was significantly fatter, which could have caused an analytical problem.

## Materials

All of the reagents (n-hexane & diethyl ether by Merck, Germany, ammonium acetate & 2-propanol by POCH, Poland) were HPLC pure. Methanol and acetonitrile (Merck, Germany) were HPLC-grade pure. HPLC-grade water was obtained from deionized water passed through a Milli-Q Gradient A10 (18.2 MV cm) water purification system (Millipore, Bedford, MA, USA). Standards of bisphenol A, 4-*tert*-octylphenol and 4-nonylphenol by SIGMA-ALDRICH^®^ USA were of high purity (>97%). Standards for the preparation of a calibration curve, in the following concentrations (10, 25, 50, 75, 100 ng cm^−3^) were prepared in methanol. Nitrogen gas was 99.995% pure (Air Liquide).

Glass vessels were used, suitably prepared by etching with nitric acid (V) (POCH, Poland) at a concentration of 3.5 mol dm^−3^ for 24 h, then being washed three times with deionized water and dried at 160 °C. The procedure for preparing the glass vessels took about 48 h. It was not possible to completely eliminate all synthetic parts - therefore e.g. blood collection vessels, pipette tips, and centrifugal vessels were checked for possible contamination during the analytical procedure.

## Sampling

Upon collection, the samples of herring were first frozen and then homogenized (as whole fish). They were then lyophilized by lyophilizer (Alpha 1-4 lD plus, Poland) for 72 h and homogenized again. Samples of herring were weighed and measured prior to freezing, and then weighed again after lyophilization, in order to determine sample wetness.

Milk was collected from female seals during lactation into 10 ml plastic tubes. Blood was collected from the seals' main lumbar vein into 3 ml plastic tubes (Profilab; Poland) with a reagent preventing blood coagulation (atomized K2EDTA). In order to minimize any risk of contamination from the vessels, blood and milk samples were frozen immediately after collection and transported to the laboratory for prompt analysis.

## Method I. Determination of BPA, OP and NP in herring (whole body)

In order to assay BPA, OP and NP in fish, a 0.1 g sample was taken and extracted with a mixture of methanol (8 cm^3^), NH_4_COOH (0.01 mol dm^−3^ 2 cm^3^) and HClO_4_ (VII) (100 μl) in an ultrasonic bath (10 min., 20 °C) (Grant Scientific XUBA3 Analogue Ultrasonic Baths; England). The samples were then centrifuged for 10 min at 3500 rpm (MPW-350R; Poland) at which point the organic layer was removed and added to 10 ml NH_4_COOH (0.01 mol dm^−3^). Extracts were purified on Oasis HLB glass cartridges (5 ml/200 mg) (Waters), optimized for trace analysis at parts-per-trillion (PPT) level. Each batch is tested for the presence of bisphenol A and phenols ([3] http://www.waters.com/waters/partDetail.htm?partNumber=186000683&locale=en_PL). We also tested the C18 SPE cartridges beforehand, as recommended by many authors e.g. Xiao et al [1]. However, they brought too high a "background" to bisphenol A.

The Oasis HLB glass cartridges were placed in a Bakerbond BAKER SPE system (Witko; Poland). Prior to use, the columns were first conditioned with 10 ml of methanol (in two 5 ml portions), and then rinsed through with 5 ml of deionized water and 5 ml of 0.01 mol dm^−3^ ammonium acetate. The sample solution was then loaded to the cartridge, washed with 5 ml of water (residual water was removed by placing the cartridge under vacuum for 30 s) and eluted with 2 × 4.0 ml of methanol at a low flow rate (1 ml/min). The eluting solution was evaporated to dryness by means of a rotary-vacuum evaporator (Heidolph Hei-VAP Advantage; Germany) at 45 ± 1 °C and reconstituted with 200 μl of acetonitrile for HPLC analysis.

## Method II. Determination of BPA, OP and NP in milk samples

The determination of BPA, OP and NP concentrations in seal milk was conducted using the method described by Yi et al. [2]. For this purpose, 2 ml of defrosted milk was incubated for 5 h at 37 degrees Celsius with an addition of 120 μl (2 M) ammonium acetate. Samples were then extracted with 4 ml of 2-propanol and centrifuged for 10 min at 3500 rpm (MPW-350R; Poland), after which the organic layer (3 ml) was removed, evaporated to dryness in the rotary-vacuum evaporator (Heidolph Hei-VAP Advantage; Germany) at 45 ± 1 °C and reconstituted with 200 μl of acetonitrile for HPLC analysis.

## Method III. Determination of BPA, OP and NP in blood samples

Bisphenol A, 4-tert-octylphenol and 4-nonylphenol in blood were assayed according to a method described by Xiao et al. [1], in which defrosted samples (0.5 ml) were extracted with a 4 ml mixture of *n*-hexane and diethyl ether (70:30 v/v) and with 100 μl of ammonium acetate (0.01 M). The samples were then centrifuged for 10 min at 3500 rpm (MPW-350R; Poland) and the organic layer was evaporated to dryness under gentle nitrogen flow. The samples were reconstituted with 200 μl of acetonitrile for HPLC analysis.

## HPLC-FL analysis

Chromatographic analysis of BPA, OP and NP was conducted using liquid chromatography with a Dionex chromatograph and UltiMate™ 3000 Fluorescence Detector (set at an excitation wavelength of 275 nm and an emission wavelength of 300 nm). Chromatographic separation was performed using a HYPERSIL GOLD C18 PAH column (Thermo Scientific) (250 × 4.6 mm; 5 μm particle size), with a mobile phase (acetonitrile and water) in gradient conditions (Table 1). The total run time was 25 min., the flow rate was 1 ml min^−1^ and the column temperature was 25 °C. The sample injection volume was 20 μl.

**Table 1:** Chromatographic separation conditions.

Time (min)	H_2_O (%)	CH_3_CN (%)
0	70	30
0	70	30
12	35	65
17	0	100
21	0	100
21,3	70	30
25	70	30

## Validation

The linearity of the methods (for blood and milk samples) was assessed by injecting different concentration levels within the range of 10–100 ng  cm^−3^. Calibration curves showed, in all cases, correlation coefficients (r) greater than 0.999. Method accuracy (estimated by means of recovery experiments in spiked samples) and precision (expressed as repeatability, in terms of Relative Standard Deviation (RSD)) were evaluated in samples spiked with a known amount of the standard for the herring, blood and milk samples. The experiments were performed in triplicate (n = 5) for each matrix. Satisfactory recovery values between 82% and 94%, with RSD lower than 15%, were obtained for all of the studied phenol derivatives (Table 2).

**Table 2:** Validation parameters.

	Method I	Method II			Method III	
	Nehring et al. [4]	Xiao et al [1]	Nehring et al. [4]	Yi et al. [2]	Nehring et al. [4]	Xiao et al. [1]
Compounds determined	BPA,OP,NP	BPA,NP	BPA,OP,NP	BPA	BPA,OP,NP	BPA,NP
Size of sample	0.5 g	0.5–1g	2 cm^−3^	4 cm^−3^	0.5 cm^−3^	0.5 cm^−3^
Matrix	Herring whole body	Rat liver	Seal milk	Human milk	Seal blood	Rat blood

	Method Differences					
Detector Fl Excitation Emission	275 nm 300 nm	227 nm 313 nm	275 nm 300 nm	225 nm 305 nm	275 nm 300 nm	227 nm 313 nm
Extraction	Oasis HLB SPE	C18 SPE				

	Validation Parameters					
Linearity/ Range	>0.999 10–100 ng cm^−3^	>0.999 10–50,000 ng cm^−3^	>0.999 10–100 ng cm^−3^	>0.999 1–120 ng cm^−3^	>0.999 10–100 ng cm^−3^	>0.999 10–50,000 ng cm^−3^
Precision	<15%	<7%	<10%	<15%	<10%	<8%
Accuracy (Recovery)	BPA 89% OP 92% NP 89%	BPA 88% NP 85%	BPA 82% OP 94% NP 84%	BPA 81%	BPA 88% OP 91% NP 83%	BPA 89% NP 87%
Quantification (LOQ) or Detection (LD) Limit	BPA 2 ng g^−1^ OP 0.8 ng g^−1^ NP 1.0 ng g^−1^ (LOQ)	BPA 1.4 ng g^−1^ NP 2.8 ng g^−1^ (LD)	BPA 0.1 ng cm^−3^ OP 0.1 ng cm^−3^ NP 0.1 ng cm^−3^ (LOQ)	BPA 1.8 ng cm^−3^ (LOQ)	BPA 0.07 ng cm^−3^ OP 0.07 ng cm^−3^ NP 0.07 ng cm^−3^ (LOQ)	BPA 2.8 ng cm^−3^ NP 5.6 ng cm^−3^ (LD)

Bisphenol A (BPA), 4-*tert*-octylphenol (OP) and 4-nonylphenol (NP).

The limit of quantification (LOQ) was estimated as a tenfold signal-to-noise (S/N) ratio from the sample chromatograms at the lowest validation level tested (n = 3) and among the studied phenols it amounted to <2 ng g^−1^ for herring, <0.07 ng cm^−3^ in blood and <0.1 ng cm^−3^ in milk.

The background (lab procedural blanks) was monitored by carrying out checks on the plastic vessels used for sampling (blood) and the centrifuge (at a frequency of every 20 uses), as well as being checked every time a new batch of SPE columns was used. The obtained “background” values for BPA, NP and OP were all < LOQ.

## Comparison of methods

The methods developed by Kim et al (2010) and by Wu et al (2006) were used with success for a wider range of phenol derivatives. The validation parameters obtained by us for the analyzed compounds, such as linearity, precision and accuracy are at a similar, satisfactory level. The limit of quantification (LOQ) obtained by us, however, is considerably lower. We used very Sensitive Fluorescence Performance. The fluorescence detector with UltiMate™ 3000 by Dionex, allowed for very low limits of detection with a Raman S/N: >550 ASTM (>2100 using dark signal as noise reference). Additionally, as the detector collects data at 200 Hz, thereby providing high sensitivity and selectivity, it enables the generation of narrow peaks and ensures high peak separation ([5] https://www.thermofisher.com/order/catalog/product/5078.0020). We also used different excitation and emission wavelength, which could have affected the sensitivity of the method (Table2).
